# Focal Adhesion Kinase Regulates Hepatic Stellate Cell Activation and Liver Fibrosis

**DOI:** 10.1038/s41598-017-04317-0

**Published:** 2017-06-22

**Authors:** Xue-Ke Zhao, Lei Yu, Ming-Liang Cheng, Pulin Che, Yin-Ying Lu, Quan Zhang, Mao Mu, Hong Li, Li-Li Zhu, Juan-Juan Zhu, Meng Hu, Po Li, Yue-Dong Liang, Xin-Hua Luo, Yi-Ju Cheng, Zhi-Xiang Xu, Qiang Ding

**Affiliations:** 1 Department of Infectious Diseases, The Hospital Affiliated to Guizhou Medical University, Guiyang, Guizhou China; 2 Department of Medicine, University of Alabama at Birmingham, Birmingham, Alabama USA; 3 Prenatal Diagnostic Center, The Hospital Affiliated to Guizhou Medical University, Guiyang, Guizhou China; 4 Comprehensive Liver Cancer Center, 302 hospital, Beijing, China; 5 Blood Transfusion Department, The Baiyun Hospital Affiliated to Guizhou Medical University, Guiyang, Guizhou China; 6 Department of Pathology, The Hospital Affiliated to Guizhou Medical University, Guiyang, Guizhou China; 7 Department of Infectious Diseases, Public Health Center of Guiyang, Guiyang, Guizhou China; 8 Department of Infectious Diseases, People’s Hospital of Guizhou province, Guiyang, Guizhou China; 9 Department of Respiratory Medicine, The Hospital Affiliated to Guizhou Medical University, Guiyang, Guizhou China; 10 Comprehensive Cancer Center, University of Alabama at Birmingham, Birmingham, Alabama USA

## Abstract

Understanding the underlying molecular mechanisms of liver fibrosis is important to develop effective therapy. Herein, we show that focal-adhesion-kinse (FAK) plays a key role in promoting hepatic stellate cells (HSCs) activation *in vitro* and liver fibrosis progression *in vivo*. FAK activation is associated with increased expression of α-smooth muscle actin (α-SMA) and collagen in fibrotic live tissues. Transforming growth factor beta-1 (TGF-β1) induces FAK activation in a time and dose dependent manner. FAK activation precedes the α-SMA expression in HSCs. Inhibition of FAK activation blocks the α-SMA and collagen expression, and inhibits the formation of stress fibers in TGF-β1 treated HSCs. Furthermore, inhibition of FAK activation significantly reduces HSC migration and small GTPase activation, and induces apoptotic signaling in TGF-β1 treated HSCs. Importantly, FAK inhibitor attenuates liver fibrosis *in vivo* and significantly reduces collagen and α-SMA expression in an animal model of liver fibrosis. These data demonstrate that FAK plays an essential role in HSC activation and liver fibrosis progression, and FAK signaling pathway could be a potential target for liver fibrosis.

## Introduction

Normal tissue repair and remodeling is a tightly controlled and self-limited process. However, persistent tissue repair and remodeling due to chronic liver diseases often lead to progressive liver fibrosis without effective treatment^1–4^. The progression and resolution of liver fibrosis are likely simultaneous processes, involving parenchymal and non-parenchymal cells, activation and apoptosis of the key fibrogenic effector cells, hepatic stellate cells (HSCs), inflammation, pro-fibrotic and anti-fibrotic/resolving immune responses, abnormal autophagy, and hepatocyte death and survival^5–9^. Once the balance is toward pro-fibrotic, excessive extracellular matrix (ECM) proteins are accumulated in liver and liver fibrosis progresses. A better understanding of the molecular mechanism(s) contributing to the progression and resolution of fibroproliferative process in chronic liver diseases will ultimately lead to the identification of novel molecular targets.

Focal adhesion kinase (FAK) is a non-receptor cytoplasmic protein tyrosine kinase and activated when cells bind to ECM proteins through integrin receptors^10–14^. Integrins are major adhesion receptors across cell plasma membrane and transmit signals between ECM ligand binding sites and their cytoplasmic domains^15^. FAK contains the N-terminal FERM (band four point one, ezrin-radixin-moesin) domain, a central kinase domain, and the C-terminal non-catalytic domain^10^. The FERM domain of FAK binds to the β-integrin subunit promoting growth factor-stimulated cell motility^10, 16^. The C-terminal domain of FAK contains several protein-protein interaction sites, and the focal-adhesion-targeting domain (FAT)^10, 17^. The FAT domain binds integrin-associated proteins (such as talin and paxillin), and directs FAK to focal adhesion complexes, which is necessary to regulate FAK-dependent cell migration^14, 18, 19^. FAK activation results in an increased autophosphorylation of Tyr397 (Y397) of FAK, a site critical for FAK to recruit downstream signaling proteins and to promote cell migration and invasion^10–12, 20^.

During chronic liver diseases, HSCs, and possible portal fibroblasts, are activated, migrate and invade into the wounded area, proliferate, and differentiate into myofibroblasts^1, 2, 21–25^. Activated FAK promotes cell migration and invasion, and mediates myofibroblast differentiation and resistance to apoptosis^10, 26–28^, suggesting a potential role for FAK in the pro-fibrotic actions of HSCs and in liver fibrosis. Transforming growth factor beta-1 (TGF-β1) is the most potent pro-fibrotic cytokine identified to date, and is currently accepted as a central mediator of the fibrotic responses in liver, lung, and kidney^3, 29–31^. Bidirectional cross-talk between integrins and ECM proteins is critical for myofibroblast differentiation, and in turn, for activation of latent TGF-β1^30, 32–34^, in which FAK mediated signaling is likely to play a direct role.

We undertook this study to investigate the functional role of FAK in HSC activation, migration, and survival, and in the development of *in vivo* liver fibrosis. The results demonstrate that FAK regulates fibrotic actions through promoting HSC activation, myofibroblast differentiation, cell migration, survival, as well as ECM protein expression. Importantly, pharmacologic inhibition of FAK attenuates liver fibrosis *in vivo* in a mouse model of liver fibrosis, suggesting that targeting FAK mediated signaling is likely to attenuate the progression of liver fibrosis.

## Results

### FAK activation is increased in fibrotic liver tissues; increased FAK activation is associated with increased expression of collagen and alpha-smooth muscle actin (α-SMA) in fibrotic liver tissues

**Figure 1:**
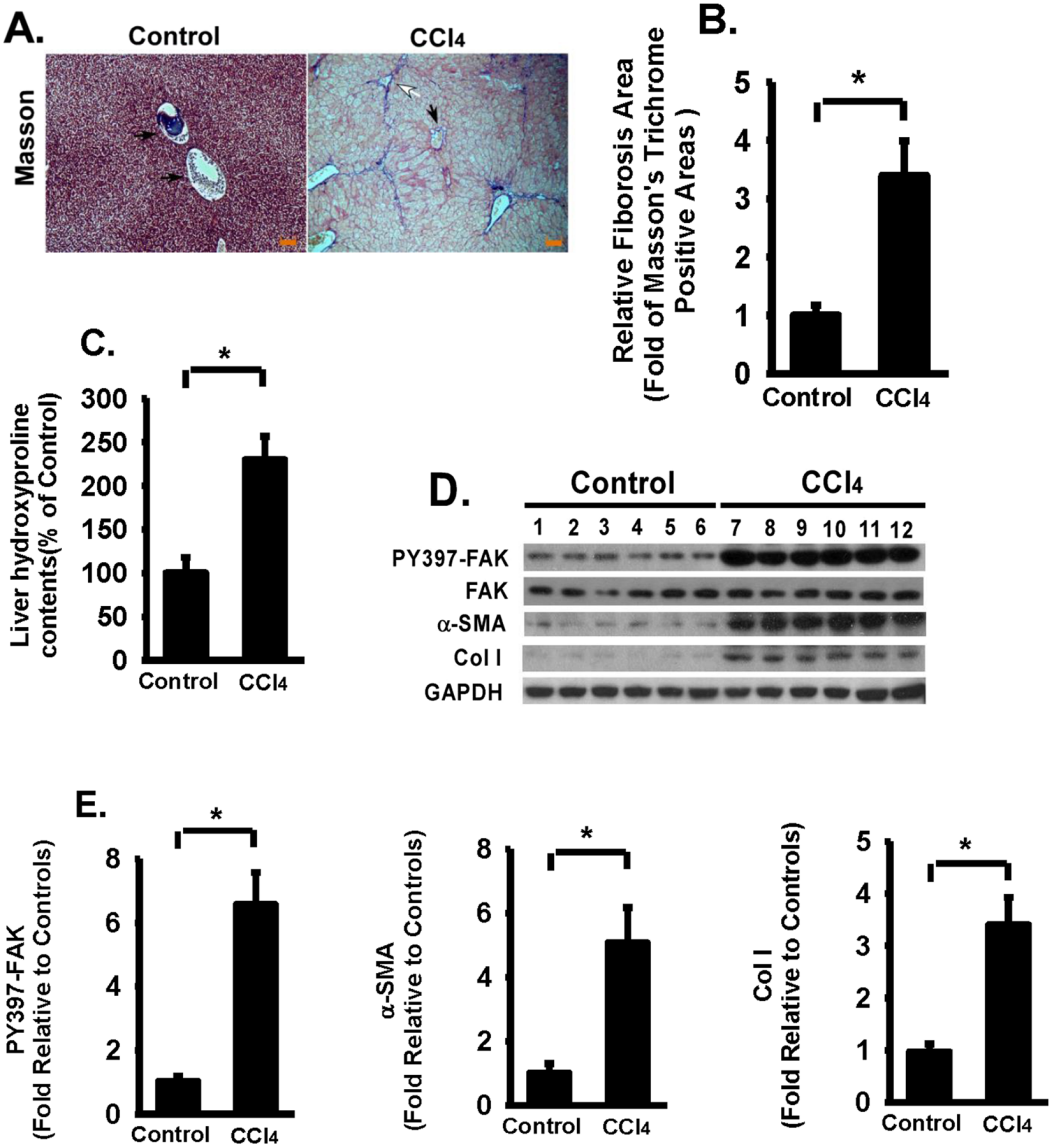
FAK activation is increased in fibrotic liver tissues; increase FAK activation is associated with increased collagen and alpha-smooth muscle actin (α-SMA) expression in fibrotic liver tissues. Mice were challenged by intraperitoneal injections of carbon tetrachloride (CCl_4_) or control vehicle (Control, corn oil as the vehicle) as described in Materials and Methods. The severity of liver fibrosis was evaluated by using several measures, including (**A**) Masson’s trichrome staining of fibrotic areas (by collagen deposition, 100X), black arrow indicates the central vein, white arrow indicates the portal vein, right bottom shows a scale bar (100 µm). (**B**) Morphometric analysis of fibrotic lesions in liver, and (**C**) Hydroxyproline levels (for collagen levels) of livers from CCl_4_ or control vehicle challenged mice. (**D**) Equivalent amount of whole liver detergent lysates were western blotted for the phosphorylation of Y397 of FAK (FAK activation), α-smooth muscle active (α-SMA, a marker for myofibroblast differentiation), or procollagen 1α1 (Col 1). Each lane represents one individual mouse. GAPDH was used as a loading control. (**E**) Densitometry analysis of phosphorylation of Y397 of FAK (normalized to total FAK), and expression of α-SMA and procollagen 1α1 (Col 1). Data were pooled and represented as mean ± SE, n = 8 animals per group. *p < 0.01.

To understand the role of FAK in the progression of liver fibrosis, we first examined the activation of FAK through phosphorylation of tyrosine 397 (Y397) of FAK in fibrotic and control liver tissues. Phosphorylation of Y397 of FAK is required for FAK activation and signaling^10^. Mice were challenged with intraperitoneal injections of carbon tetrachloride (CCl_4_) or control vehicle (corn oil); hepatic fibrosis in mice induced by carbon tetrachloride is a widely used mouse model to study liver fibrosis^35, 36^. Using several measures, the fibrotic response was greatly increased in to carbon tetrachloride treated mice when compared to control mice. Live fibrosis were confirmed by Masson’s trichrome staining (Fig. 1A), morphometric analysis of fibrotic lesions (Fig. 1B), and hydroxyproline levels (a surrogate for the increased collagen deposition, Fig. 1C) in carbon tetrachloride treated mice.

Importantly, phosphorylation of Y397 of FAK (an indication of FAK activation), not total FAK protein, was significantly increased (about 6 fold increase) in fibrotic liver tissues when compared to control liver tissues (Fig. 1D and E). Meantime, α-SMA (about 5 fold, Fig. 1D and E) and collagen (about 3.5 fold, Fig. 1D and E) expression were significantly increased. The data demonstrate that increased FAK activation is associated with increased ECM production (collagen) and one critical marker of hepatic stellate cells activation (α-SMA).

### Transforming growth factor beta-1 (TGF-β1) induces FAK activation in a dose- and time-dependent manner in primary hepatic stellate cells (HSCs); FAK activation precedes α-SMA expression in HSCs

**Figure 2:**
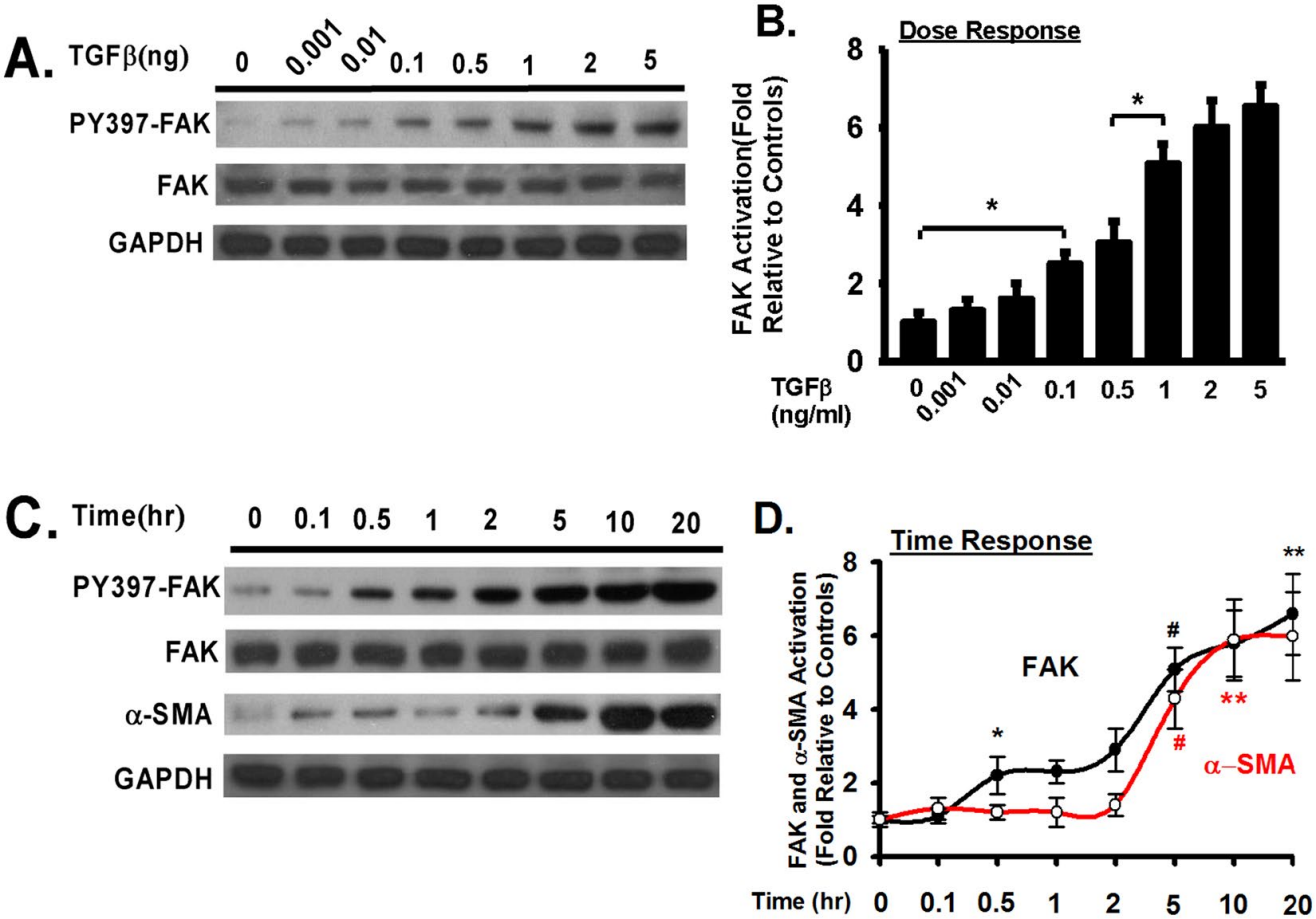
Transforming growth factor beta-1 (TGF-β1) induces FAK activation in a dose- and time-dependent manner in primary hepatic stellate cells (HSCs); FAK activation precedes α-SMA expression in HSCs. (**A**) Primary mouse HSCs were serum starved for 20 hours, treated with TGF-β1 at the indicated dose (ng/ml) for 24 hours, and lysed. Equivalent amount of whole cell detergent lysates were western blotted with the indicated antibodies for phosphorylation of Y397 of FAK (FAK activation), total FAK, or GAPDH. (**B**) Densitometry analysis of phosphorylation of Y397 of FAK (normalized to total FAK) at the indicated TGF-β1 dose. (**C**) Primary mouse HSCs were serum starved for 20 hours, treated with TGF-β1 (2 ng/ml) for the indicated time periods (hour, hr), and lysed. Equivalent amount of whole cell detergent lysates were western blotted with the indicated antibodies. (**D**) Densitometry analysis of the association of phosphorylation of Y397 of FAK (normalized to total FAK) and α-SMA at the indicated time in response to TGF-β1 stimulation. Data were pooled from at least three independent experiments and represented as mean ± SE. *p represents < 0.05. # or **represents < 0.01 when compared to time 0.

TGF-β1 is considered as one of the most important pro-fibrotic cytokine in many organ fibrosis^37^. We next examined the FAK activation pattern in primary mouse HSCs (derived from C57BL6 mice) in response to TGF-β1. The basal (without challenge) FAK activation (through phosphorylation of Y397 of FAK) is minimal in serum starved HSCs (Fig. 2A). FAK activation was increased in a dose-dependent manner in response to TGF-β1 stimulation, with apparent two peaks, one small peak at 0.1 ng/ml and another strong peak at 1 ng/ml of TGF-β1 in HSCs (Fig. 2A and B). FAK was clearly activated as early as 30 minutes (at 2 ng/ml dose of TGF-β1), and FAK activation is significantly at 5 hours after TGF-β1 stimulation (2 ng/ml) (Fig. 2C and D). Meanwhile, α-SMA expression was clearly increased at 5 hours after TGF-β1 stimulation (2 ng/ml) (Fig. 2C and D). The increase of FAK activation precedes the increase of α-SMA expression in TGF-β1-treated primary mouse HSCs, suggesting that FAK may play a role in TGF-β1-induced α-SMA expression.

### FAK activation is necessary for HSC activation, myofibroblast differentiation, and collagen expression induced by TGF-β1

**Figure 3:**
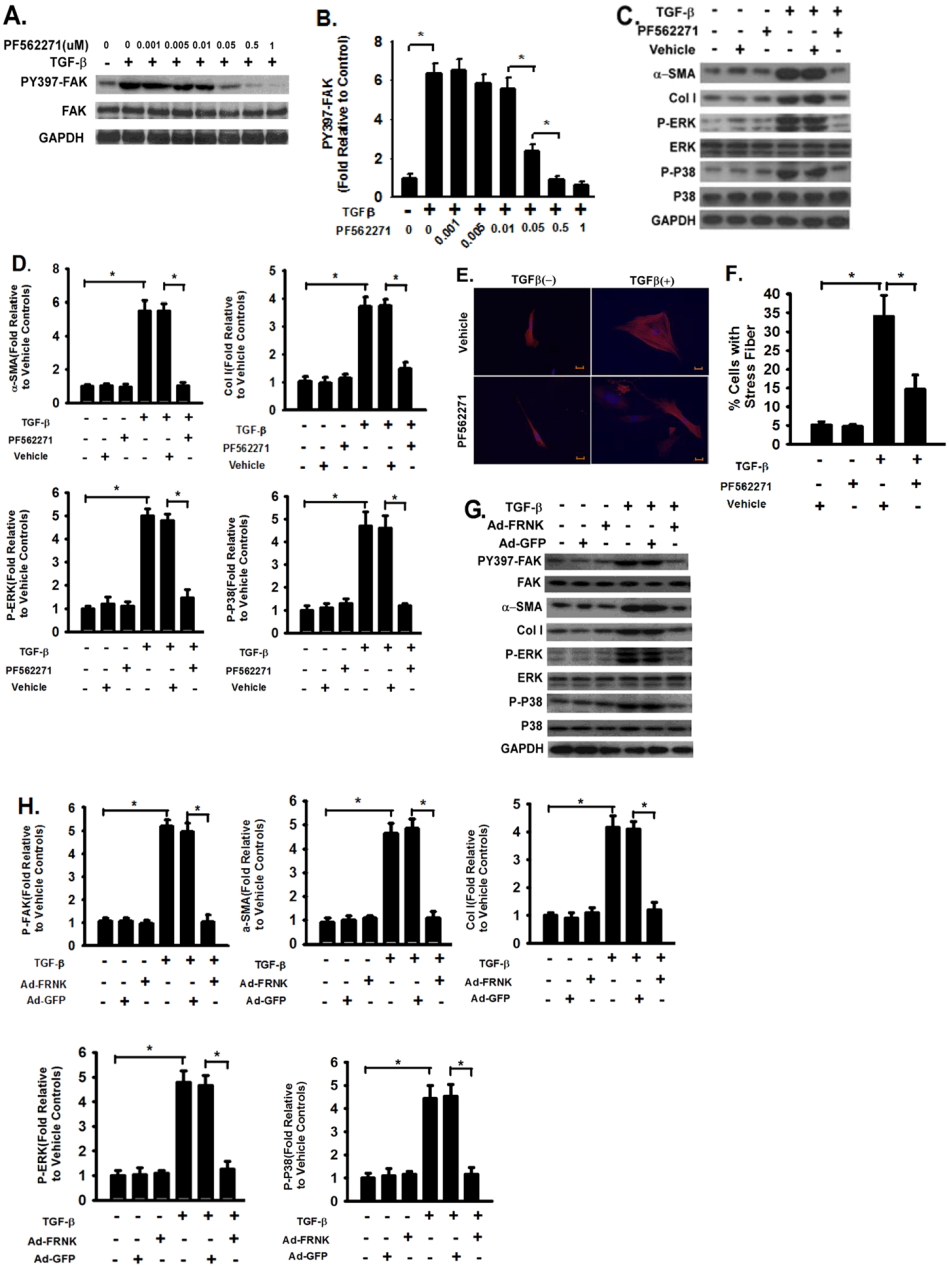
Pharmacologic inhibition of FAK activation inhibits HSC activation, myofibroblast differentiation, and collagen expression. (**A**) Serum starved (for 20 hours) primary mouse HSCs were treated with TGF-β1 (2 ng/ml) followed by FAK inhibitor (PF562271) at the indicated dose or vehicle for 24 hours, and lysed. Equivalent amount of whole cell detergent lysates were western blotted with the indicated antibodies. (**B**) Densitometry analysis of the effect of FAK inhibitor on phosphorylation of Y397 of FAK (normalized to total FAK) at the indicated dose. (**C**) Serum starved (for 20 hours) primary mouse HSCs were treated with TGF-β1 (2 ng/ml) followed by FAK inhibitor (PF562271, 0.5 µM) or vehicle for 24 hours, and lysed. Equivalent amount of whole cell detergent lysates were western blotted with the indicated antibodies. (**D**) Densitometry analysis of the effect of FAK inhibitor on myofibroblast differentiation (by α-SMA expression) and collagen expression (Col 1) (normalized to GAPDH) in panel C. (**E**) HSCs were treated as in panel C, fixed after 24 hours, immunofluorescently stained with Cy-3-labelled monoclonal antibody toward α-SMA (red), and fluorescent microscopic digital images were taken (200X). Nuclear were staining with Hoechst (blue) and right bottom shows a scale bar (15 µm). Representative pictures were shown. (**F**) Quantification of the percentage of cells with newly formed α-SMA-containing stress filaments. (**G**) Cells were infected with adenoviral vectors containing the dominant negative regulator of FAK, the FAK-related non-kinase (FRNK) or GFP and treated as in panel C. Equivalent amount of whole cell detergent lysates were western blotted with the indicated antibodies. (**H**) Densitometry analysis of the effects of FRNK expression in panel G. Data were pooled from at least three independent experiments and represented as mean ± SE. *p represents < 0.01.

The pharmacologic approach was used to investigate the role of FAK in HSC activation. Small molecule FAK inhibitor (PF562271) blocked TGF-β1-induced FAK activation (phosphorylation of Y397 of FAK) in primary mouse HSCs in a dose-dependent manner (Fig. 3A). TGF-β1-induced FAK activation was completely inhibited at 1 µM dose of FAK inhibitor (Fig. 3A and B). Inhibition of FAK activation almost completely blocked TGF-β1-induced expression of α-SMA and collagen in primary mouse HSCs (Fig. 3C, lane 6 versus 4, and Fig. 3D). Inhibition of FAK activation also attenuated TGF-β1-induced activation of extracellular signal–regulated kinase (ERK) and p38 mitogen-activated protein kinase (Fig. 3C, lane 6 versus 4, and Fig. 3D). To validate and confirm the findings of pharmacologic approach, we used another genetic approach by overexpressing a dominant negative regulator of FAK, the FAK-related non-kinase (FRNK, a dominant negative 3′ region transcripts against FAK) as previously described^26^. FRNK inhibited TGF-β1-induced FAK activation in HSCs (Fig. 3G and H). FRNK also blocked the expression of α-SMA and collagen, and attenuated the activation of ERK and p38 (Fig. 3G and H) demonstrating that the inhibitory effects of FAK inhibitor on HSCs are specific. During HSC activation and myofibroblast transition, there are profound cytoskeleton changes, including the newly formed thick and straight stress fibers^5, 21–23^. Inhibition of FAK activation blocked the cytoskeleton reorganization and formation of stress fibers in TGF-β1-treated primary mouse HSCs (Fig. 3E and F), supporting that FAK activation is necessary for HSC activation and myofibroblast transition.

### FAK promotes platelet derived growth factor BB (PDGF-BB) stimulated HSC migration through small Rho GTPases (Rac and Rho)

**Figure 4:**
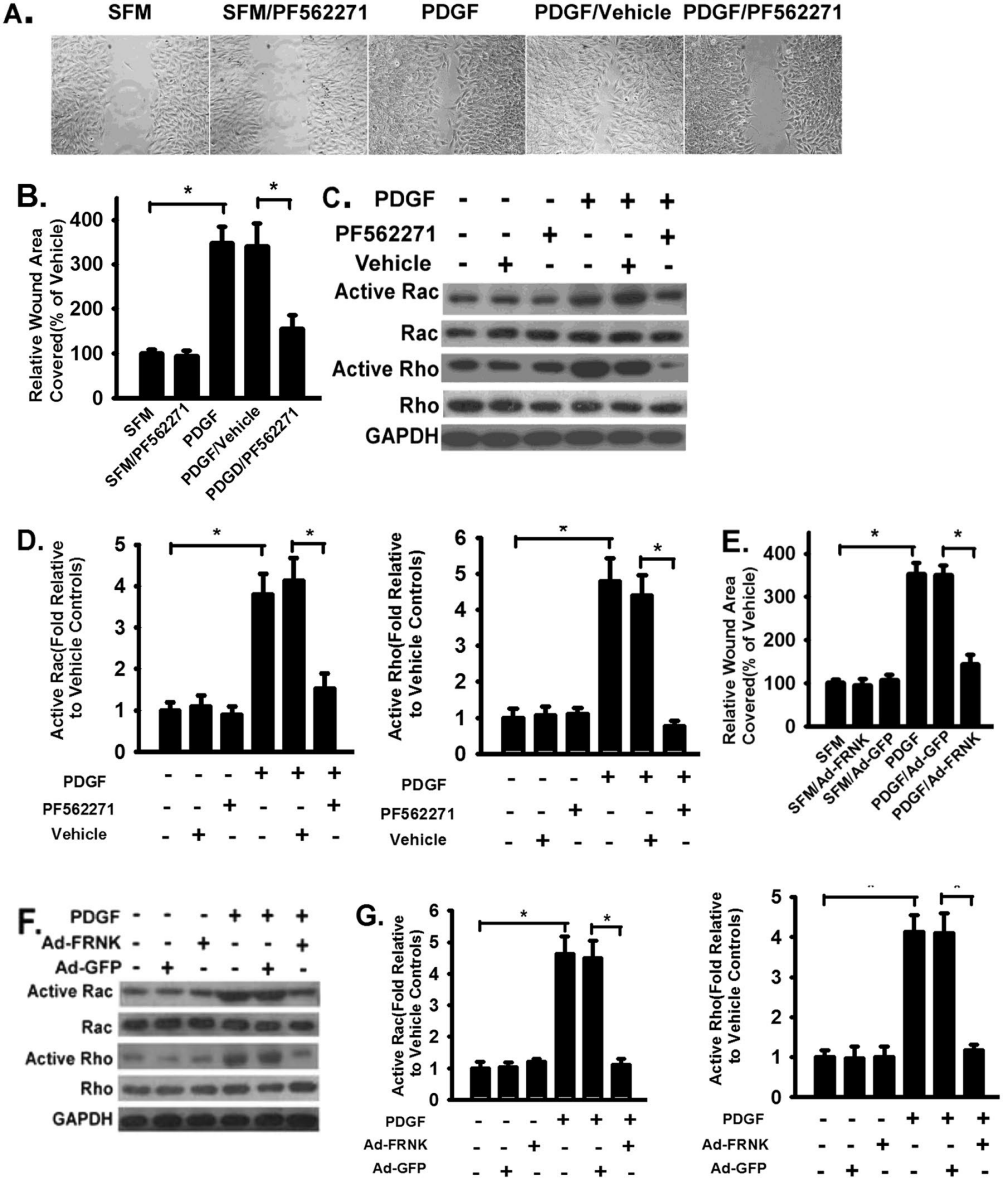
FAK inhibition significantly reduces HSC migration and inhibited the activation of Rac and Rho in response to platelet derived growth factor BB (PDGF-BB). (**A**) Primary mouse HSCs were serum starved for 20 hours and HSC migration in response to PDGF-BB (PDGF, 4 ng/ml) in serum-free medium with 1% BSA (SFM) with FAK inhibitor (PF562271, 0.5 µM) or vehicle was examined by wound closure assay for 24 hours. Representative images were shown (10X). Migration in SFM condition was almost not observed. Image at 0 in SFM (first image) was used to quantify the migration. Other images were taken at 24 hour for the indicated conditions. (**B**) Data are pooled and shown as % of wound area covered by cells over 24 hours, relative to that of uninfected WT fibroblasts in SFM medium. (**C**) HSCs were treated as in panel A and lysed at 24 hours. Equivalent amount of whole cell detergent lysates were used for detection of Rac and Rho activation. (**D**) Densitometry analysis of the effect of FAK inhibitor on Rac and Rho activation at the indicated condition. (**E**) Cells were infected with adenoviral vectors containing FRNK or control GFP, treated as in panel A, and migration was examined by wound closure assay for 24 hours after PDGF-BB (PDGF, 4 ng/ml) treatment. Data were pooled and represented as in panel B. (**F**) HSCs were treated as in panel E and lysed at 24 hours for activation of Rac and Rho. (**G**) Densitometry analysis of the effect of FRNK expression on Rac and Rho activation at the indicated condition. Data were pooled from three independent experiments and represented as mean ± SE. *p represents < 0.01.

HSC migration contributes to the development of liver fibrosis. The role of FAK in HSC migration was examined by 2D wound healing assays. Platelet growth factor BB (PDGF) is a potent cytokine inducing cell migration in multiple cell types^38^. PDGF induced primary mouse HSC migration into wound areas (Fig. 4A) and FAK inhibition (by PF562271) blocked the PDGF-induced HSC migration (Fig. 4A and B). Basal migration in serum free (SFM, 1%BSA) was minimal or almost not observed (data not shown). Small Rho GTPase plays a critical role in cell migration through modulation of focal adhesion and cytoskeleton^39, 40^. PDGF stimulation induced activation of small Rho GTPases. Rac and Rho activation was increased in PDGF-treated HSCs (Fig. 4C, lane 4 versus lane 1); FAK inhibition significantly reduced PDGF-induced Rac and Rho activation (Fig. 4C and D). Similar to the effects of FAK inhibitor, FRNK overexpression inhibited PDGF-induced HSC migration (Fig. 4E) and activation of Rac and Rho (Fig. 4F and G).

### FAK plays an important role in survival of activated HSCs

**Figure 5:**
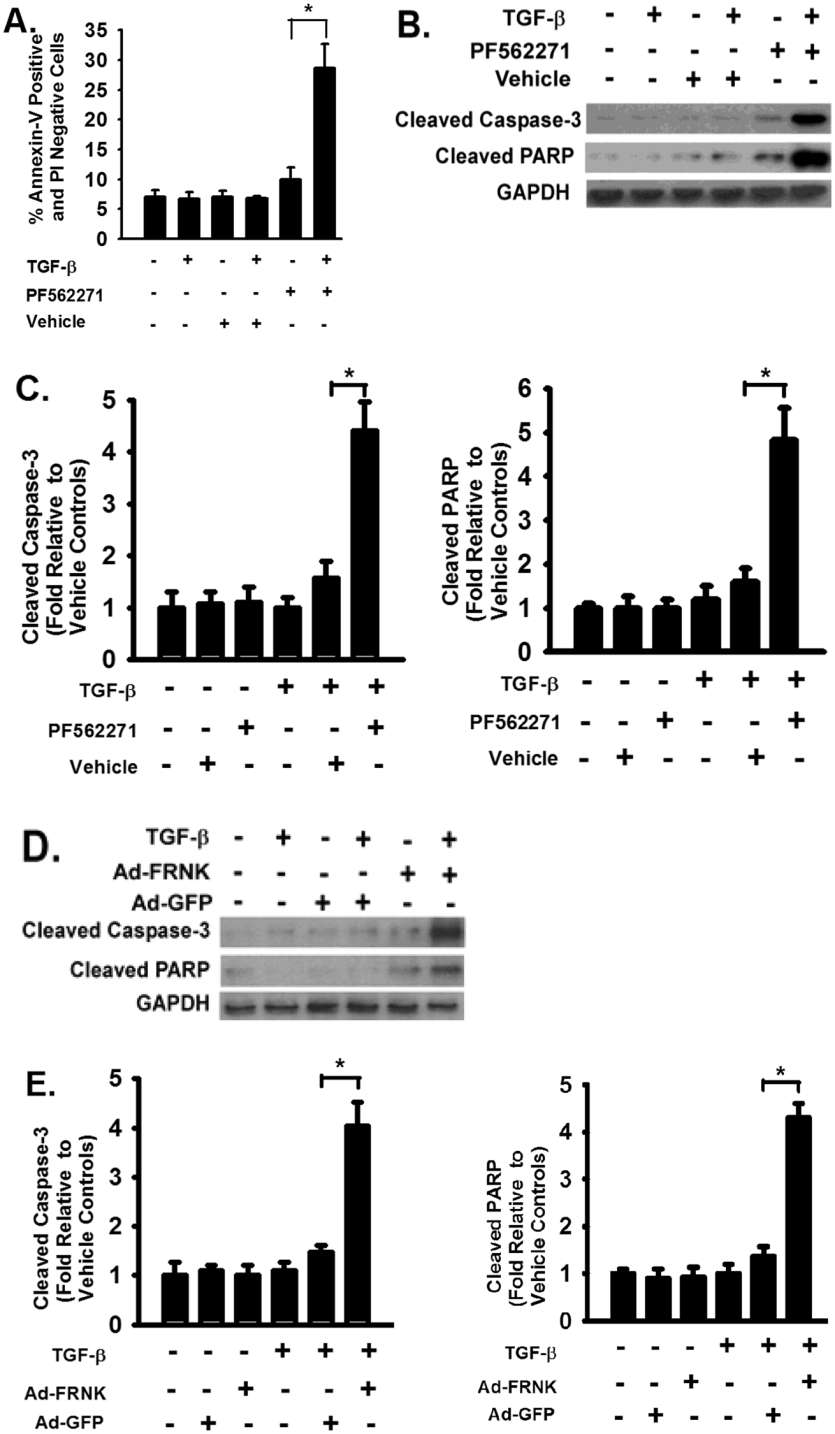
FAK inhibition induces apoptotic signaling in activated HSCs. (**A**) Primary mouse HSCs were treated with TGF-β1 (2 ng/ml) followed by FAK inhibitor (PF562271, 0.5 µM) for 24 hours and subject to Annexin-V and PI labeling apoptosis assays as described in Materials and Methods. The percentage of early apoptotic cells were marked by Annexin-V-positive and PI-negative. (**B**) HSCs were treated as in panel A and lysed at 24 hours. Equivalent amount of whole cell detergent lysates were Western blotted with antibody recognizing only cleaved caspase-3 or cleaved PARP for apoptotic signaling. (**C**) Densitometry analysis of the effect of FAK inhibitor on cleaved caspase-3 and PARP at the indicated condition. (**D**) HSCs were infected with adenoviral vectors containing FRNK or control GFP and treated as in panel A, then lysed at 24 hours followed by Western bot analysis with indicated antibodies as in panel B. (**E**) Densitometry analysis of the effect of FRNK expression on cleaved caspase-3 and PARP at the indicated condition. Data were pooled from three independent experiments and represented as mean ± SE. *p represents < 0.01.

Resistance of apoptosis of HSCs contributes to the progression of liver fibrosis. The effect of FAK inhibition on survival of primary mouse HSCs was examined. There was no significant difference notice between TGF-β1 treated HSCs and vehicle treated HSCs (Fig. 5A); however, FAK inhibition significantly increased the apoptotic cells, evidenced by that Annexin-V positive cells were significantly increased in HSCs treated with TGF-β1 and FAK inhibitor (Fig. 5A). There was a slight increase of Annexin-V positive cells in HSCs treated with FAK inhibitor alone (Fig. 4A, bar 5 versus 1). Western blot analysis confirmed that there was an increase in apoptotic signaling pathway. FAK inhibition by pharmacologic inhibitor increased the cleaved Caspase-3 and PARP (Fig. 5B and C). Similar results were obtained in HSCs expressing FRNK and FRNK overexpression increased the cleaved Caspase-3 and PARP (Fig. 5D and E), supporting that FAK plays an important role in survival of TGF-β1 activated HSCs.

### FAK inhibition attenuates liver fibrosis *in vivo*

**Figure 6:**
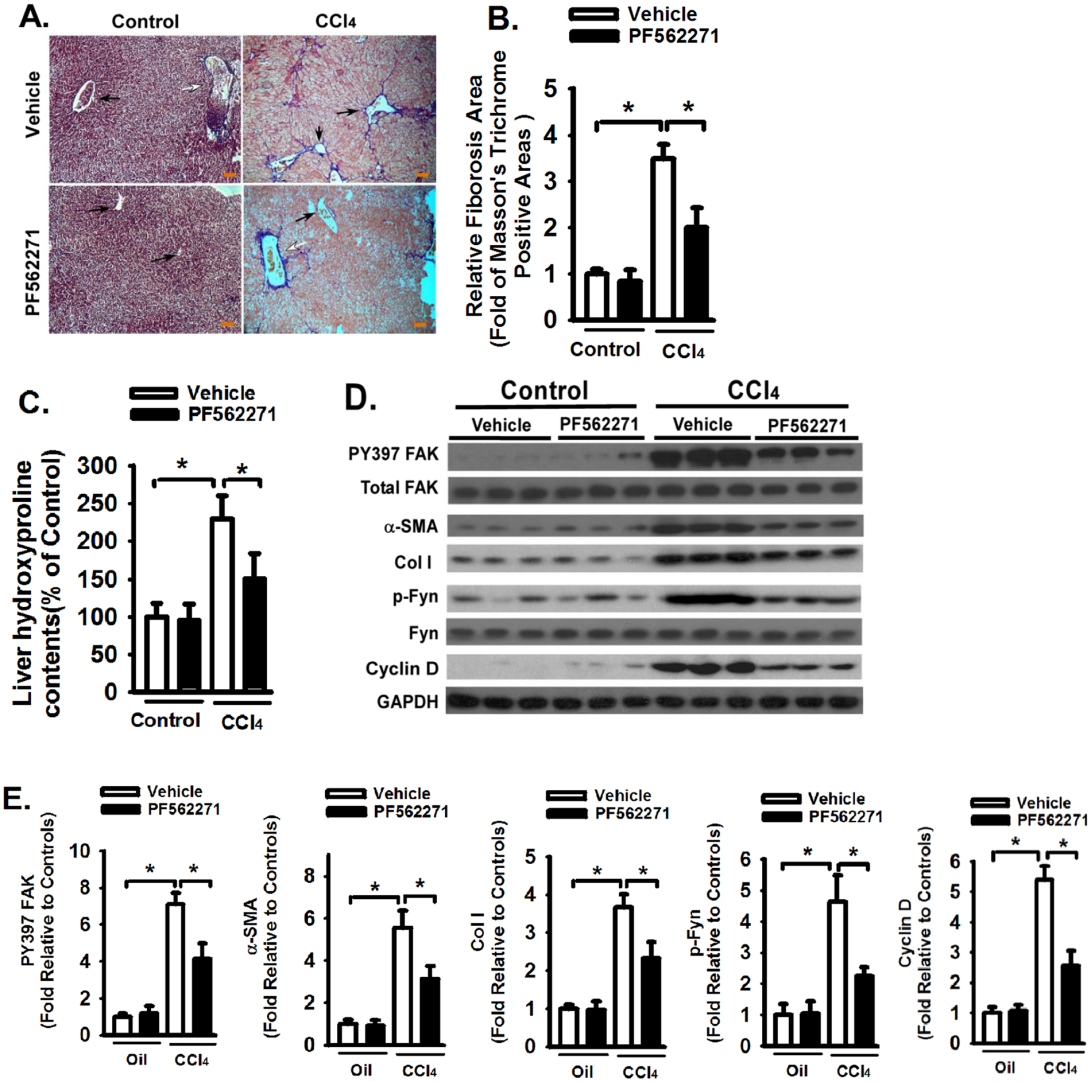
FAK inhibition attenuates liver fibrosis *in vivo*. Mice were challenged by carbon tetrachloride (CCl_4_) or corn oil (Control), followed with oral administration of FAK inhibitor (PF-562271) (30 mg/kg in 0.5% methylcellulose, daily, p.o. by gavage) or with 0.5% methylcellulose control (Vehicle) as described in Materials and Methods. The severity of liver fibrosis was evaluated (**A**) Masson’s trichrome staining (100X), black arrow indicates the central vein, white arrow indicates the portal vein and area, right bottom shows a scale bar (100 µm). (**B**) Morphometric analysis of fibrotic lesions in liver, and (**C**) Hydroxyproline levels (for collagen levels) of livers challenged mice. (**D**) Equivalent amount of whole liver detergent lysates were western blotted with the indicated antibodies. Each lane represents one individual mouse. GAPDH was used as a loading control. (**E**) Densitometry analysis of the effect of FAK inhibitor (PF562271) on FAK activation, myofibroblast differentiation (α-SMA), ECM production (Col 1), activation of Src kinase (Fyn), and proliferation signaling (Cyclin D1). Data were pooled and represented as mean ± SE, n = 8–9 animals per group. *p < 0.01.

To further understand the role of FAK in the development of liver fibrosis *in vivo*, mice were treated with intraperitoneal carbon tetrachloride (CCl_4_) injections, followed by daily treatment of FAK inhibitor (PF562271) (Fig. 6). Using several measures, FAK inhibitor significantly reduced liver fibrosis, including decreased liver collagen production and deposition (Fig. 6A and C), decreased fibrotic lesions (Fig. 6B), and decreased α-SMA expression and collagen expression in liver tissues (Fig. 6D and E). Reduced FAK activation (phosphorylation of Y397 of FAK) was confirmed in liver tissue by Western blot analysis (Fig. 6D). Interestingly, Fyn, a Src kinase family member, has shown increased activation in carbon tetrachloride treated liver tissues; while FAK inhibition significantly reduced the Fyn activation (Fig. 6D and E). It has been shown that FAK and Src kinase reciprocally activate each other^10^; therefore, Fyn may be involved in FAK activation during liver fibrosis progression. In addition, Cyclin D, a cell proliferation Cyclin, was increased in carbon tetrachloride treated liver tissues; while FAK inhibition significantly inhibited Cyclin D expression (Fig. 6D and E). Taken together, the data demonstrate that FAK plays an important role in the progression of liver fibrosis.

## Discussion

The main findings in this manuscript are that FAK plays an important role in liver fibrosis progression through modulating HSC activation and promoting TGF-β-driven pro-fibrotic responses in HSCs. These findings were substantiated *in vitro* through FAK inhibition significantly reduced TGF-β-driven HSC activation, myofibroblast differentiation, and ECM production. FAK inhibition also blocked PDGF-induced cell migration, inhibited Rac and Rho activation which are known to promote cell migration, and induced apoptotic signaling in activated HSCs. Furthermore, these findings were substantiated *in vivo* through the decrease in liver fibrosis measured at the histological, biochemical, and cell signaling levels in carbon tetrachloride-challenged mice treated with FAK inhibitor. This study implicates that FAK plays an important role in the regulation of lvier fibrosis *in vivo*, and provides evidence for the potential molecular mechanism(s) of FAK’s pro-fibrotic actions.

FAK activation is increased in fibrotic liver tissues from carbon tetrachloride-challenged mice when compared to control vehicle treated mice (Fig. 1). The increased FAK activation in fibrotic liver is likely due to multiple stimulus. FAK can be activated by integrin clustering due to binding to ECM proteins^10^. The increased ECM protein production and deposition are likely one of the reasons to see increased FAK activation in fibrotic liver versus minimal FAK activation in normal liver. FAK also can be activated by growth factors and cytokines^10^. Due to liver injury caused by carbon tetrachloride, it is expected that inflammation cytokines and growth factors are abundant in response to liver injury, such as TGF-β1, which has been to induce FAK activation^26, 41^ and it is a potent cytokine to activate HSCs^2, 5, 23^.

TGF-β is a pleiotropic cytokine, and plays a central role in fibrogenesis in multiple organs, including liver and lung^1, 42^. TGFβ1 is one best studied pro-fibrotic cytokine; it is well known for its powerful effect in inducing synthesis of ECM proteins, such as collagen and fibronectin, and in stimulating myofibroblast differentiation^3, 37, 43^. Studies have demonstrated that inhibition of TGF-β pathway by inhibitors, antibodies, deletion of its receptor, or blockade of downstream signaling (such as Smad3), reduces fibrosis^42, 44–46^. However, systemic inhibition of TGFβ1 on patients, not limited to the fibrotic milieu, likely to have some confounding side effects, such as neoplasia, since many types of human cancer have loss of function mutations on TGFβ receptors^47–50^. Therefore, in order to inhibit TGFβ driven fibrotic action to treat liver fibrosis, one could consider to block its upstream or downstream signaling proteins for antifibrotic efforts in liver.

This study suggests that FAK could be a promising target. The data reveal that TGF-β1 stimulation significantly increased FAK activation in a dose and time dependent manner (Fig. 2) and the increase of FAK activation in fibrotic liver tissue is associated with increased α-SMA and collagen production (Fig. 1). This is consistent with that TGF-β1 induces FAK activation in lung fibroblasts^26, 41^. Interestingly, the peak of FAK activation precedes the expression of α-SMA, the expression of the reliable cellular marker of HSC activation (Fig. 2). FAK inhibitor blocked TGFβ induced α-SMA and collagen production (Fig. 3). It has been reported that FAK is positively associated with α-SMA expression in hepatic fibrosis^51^. These observations clearly support that FAK signaling is required for HSC activation and myofibroblast differentiation. FAK has been shown to promote cytoskeletal reorganization during myofibroblast differentiation and inhibition of FAK reduces the stress fiber formation induced by TGF-β1^41^. It is likely that similar mechanism may be used by FAK to promote HSC activation and differentiation into myofibroblasts as FAK inhibition blocked the stress fiber formation in HSCs (Fig. 3).

The role of FAK in fibrosis could be more complicated than the current study suggests and through multiple pathways. PDGF-BB induces FAK activation^52, 53^. FAK is required for HSC migration induced by PDGF-BB and HSC survival, as FAK inhibition resulted in decreased migration and increased apoptotic signaling evidenced by increased cleaved caspase-3 and PARP (Fig. 5). The data is consistent with the observation that increased FAK activation contributes to fibroblast migration derived from human fibrotic lungs^54^ and hepatic myofibroblasts^52^. FAK inhibition also induce apoptosis in Hepatitis C virus infected LX-2 cells^55^. FAK has been shown to mediate collagen expression and antifibrotic effect of adiponectin^56^. PDGF signaling also promotes HSC proliferation through platelet-derived growth factor receptor-beta (PDGFR-β), whose expression in healthy liver is low^57, 58^. Cyclin D1 expression is increased in CCl4-induced fibrotic liver (Fig. 6), a result consistent with previous findings^59^. FAK inhibition *in vivo* reduces cyclin D1 expression (Fig. 6), likely inhibition of cell proliferation. Integrin clustering is known to activate FAK^10^ and it is involved in TGFβ1 activation as blocking integrin receptor (αvβ6) inhibits latent TGFβ1 activation^46^. FAK is a mechanosensor and modulates cytoskeletal changes for mechanical tension^10, 60–62^. Whether FAK is involved in modulation of the mechanical tension needed to activate latent TGFβ1 is unclear and will be a subject for future studies. FAK regulates fibroblast migration via integrin beta-1 and plays a central role in fibrosis^63^. If so, FAK signaling not only contribute to the TGFβ1 induced HSC activation, migration, and survival, but also contribute to the TGFβ1 activation through integrins in a feed forward manner to promote HSC activation and liver fibrosis.

It is only through a completely understanding of the molecular mechanisms contributing to hepatic fibrosis that we will be able to design more effective therapy. Our study has investigated the role of FAK in HSC activation and the progression of hepatic fibrosis by both *in vitro* and *in vivo* approaches. The data reveal that FAK promotes HSC activation, differentiation, migration, matrix synthesis, and survival. Importantly, FAK inhibition attenuates hepatic fibrosis in a CCl4 mouse model of liver fibrosis. Thus, FAK and the FAK pathway represent new therapeutic targets that may limit the progression of hepatic fibrosis.

## Materials and Methods

### Reagents and Antibodies

Transforming growth factor-β1 (TGF-β1) and platelet derived growth factor BB (PDGF-BB) were obtained from R&D Systems (Minneapolis, MN). The following antibodies were purchased: phospho-FAK [pY397] (Biosource, Camarillo, CA), procollagen alpha 1 type 1 (1A1), Fyn, Cyclin D1 (Santa Cruz Biotechnology, Santa Cruz, CA), FAK (N-terminal domain) (Santa Cruz, CA), Fyn and phospho-SrcY418 (Cell Signaling Technology, Boston, MA), phosphor-ERK and ERK, phosphor-p38 and p38, Cleaved caspase-3, cleaved Poly-(ADP-ribose) polymerase (PARP), (EMD Millipore, Billerica, MA), and glyceraldehyde 3-phosphate dehydrogenase (G3PDH) (Research Diagnostics, Flanders, NJ). Anti-α-SMA antibody, Cy3-conjugated anti-α-SMA antibody, carbon tetrachloride (CCl_4_), corn oil, and OptiPrep were purchased from Sigma-Aldrich (St. Louis, MO). FAK inhibitor (PF-562271) was purchased from Selleckchem and Fisher Scientific (Waltham, MA). Kits for active Rac and Rho were purchased from EMD Millipore (Billerica, MA). The generation and production of adenoviral vectors containing FAK-related non-kinase (ad-FRNK) or control green fluorescent protein (GFP) were described previously^26^. All other chemicals and reagents were purchased from Sigma-Aldrich (St. Louis, MO) and Fisher Scientific (Waltham, MA).

### The mouse model of liver fibrosis

All animal interventions were approved by local Institutional Animal Care and Use Committee (IACUC) at Guizhou Medical University and University of Alabama at Birmingham. The methods and experiment procedures were carried out in accordance with the relevant guidelines and regulations. Mice (C57BL6, male and female, eight to ten week old) were house in standard condition and sex matched mice were treated with 1.5 µl/g body weight carbon tetrachloride (CCl_4_) (1:10 [volume:volume] dilution with corn oil) or corn oil as control by intraperitoneal (i.p.) injections, three times per week for 4 weeks. Mice were treated with FAK inhibitor (PF-562271) by oral administration (30 mg/kg in 0.5% methylcellulose, daily, p.o. by gavage) or only methylcellulose control. Mice were sacrificed at 48 hour after the last CCl_4_ injection and tissues were harvested.

### Collagen Determination

The collagen level was determined by hydroxyproline level as described^64, 65^. The harvested liver tissues were homogenized and hydrolyzed in 6 M HCl at 110 °C for 24 hours, and the amount of hydroxyproline in the tissue acid-hydrolysates was performed by colorimetric assay as described^65^. Collagen deposition in liver tissue sections (5–10 µm, paraffin embedded tissues) was localized by Masson’s trichrome staining using a commercially available staining kit according to the manufacturer’s instructions (Poly Scientific, Bay Shore, NY). Fibrotic lesional density by area was measured on Masson’s trichrome stained sections and quantified by morphometric methodology.

### Cells and Cell Culture

Isolation of primary mouse hepatic stellate cells (HSCs) were by enzymatic digestion and density gradient centrifugation as described previously^36, 66, 67^. Briefly, cells were isolated from the livers by *in situ* liver perfusion with pronase, liberase, and collagenase followed by density gradient centrifugation. Dispersed cell suspension was filtered and centrifuged at 50 g for 2 min to remove hepatocytes. The remaining cell fraction was washed and resuspended in 11.5% OptiPrep, then gently transferred to the tube containing a bottom of 15% OptiPrep, followed by adding the top layer of PBS. The cell fraction was then centrifuged at 1400 g for 20 minutes. The HSC fraction layer was obtained at the interface between the top and intermediate layer. The purity of the HSCs fraction was estimated based on autofluorescence. Purity of HSCs was assessed and estimated by autoflorescence one day after isolation, and always was higher than 97%. Cell viability was also examined and HSCs were maintained in Dulbecco’s modified Eagle’s medium (DMEM) supplemented with 10% fetal bovine serum (FBS) and antibiotics as described previously^54^. Cells were started starvation or experiments at day 2 after isolation and the duration of starvation or treatment are described in each figure legend. The duration of the whole experiments was within 5–7 days of isolation of HSCs and the passages were 1–2 (as some experiments were needed to passage cells from regular culture flask to experimental cell culture wells).

### Western Blotting Analysis and Rac and Rho Activation Assays

Immunoblotting was performed on 1% NP-40 whole liver tissue lysates or whole cell lysates as described previously^54^. Equivalent micrograms of lysates were electrophoresed on a disulfide-reduced 8–12% SDS PAGE, transferred to Immobilon-P membrane (Millipore Corp., Bedford, MA) for probing, and developed with the enhanced chemiluminescence (ECL) system (Fisher Scientific). Rac and Rho activation were determined by the level of GTP-bound forms of Rac and Rho per the instructions (kits from Millipore, Billerica, MA) as described previously^54^.

### Cell Migration Assay

The wound closure monolayer/scratch motility assay was performed as described previously^54^. Briefly, fibroblasts were plated in serum-free DMEM with 1% BSA for 24 hours. Mitomycin C was added to inhibit cell proliferation. The monolayer was scratched, and the wound area covered by cell migration over indicated time on digital photomicrograph images was calculated.

### Immunofluorescence (IF) Analysis

IF was performed as described previously^41^. Briefly, cells cultured on glass-coverslips were fixed in 4% buffered paraformaldehyde, permeabilized. To study the α-SMA-incorporated cytoplasmic filaments, cells were reacted with Cy3-conjugated anti-α-SMA monoclonal antibody (1:50) for overnight at 4 °C. Digital fluorescence images were obtained by using a Nikon microscope and software (Nickon Inc.). The percentage of cells containing highly organized, thickened α-SMA-incorporated filaments over total cells was quantified. The results were pooled from 8 randomly selected, non-overlapping fields from a total three experiments (each performed in duplicates).

### Annexin V apoptosis assay

The Annexin V assay with propidium iodide labeling was performed with apoptosis kit (BD Pharmingen, San Diego) on pancreatic tumor cells with indicated treatment as per the manufacturer’s instructions and described previously by us^19, 68^.

### Statistical analysis

Data were analyzed using the Student’s t-test analysis (Sigma Plot, SPSS Inc.) for differences between two groups, and expressed as mean ± SE. For comparisons between multiple groups, an ANOVA (3-way ANOVA) test was performed, followed by t-tests with Bonferronni correction using SAS 9.3 (SAS Institute Inc., Cary, NC, USA). All experiments were repeated at least 3 times. A *p* value < 0.05 was considered to be statistically significant.
